# Supplementary material for: A Quick Guide to CAF Subtypes in Pancreatic Cancer
Source: Cancers (Basel). 2023 May 4;15(9):2614. doi: 10.3390/cancers15092614 (PMC10177377; doi:10.3390/cancers15092614)
Supplement: Supplementary file 1 [file cancers-15-02614-s001.zip › cancers-2331270-supplementary.pdf]

Table S1. A summary of currently known CAF subpopulations.

Subtype	Functions	References
**Tumor-restraining CAFs**		
**Myofibroblasts (myCAFs)**	• α-Sma⁺, extracellular matrix deposition<br>• Production of heterotrimeric collagen I<br>• Efficient recruitment and activation of T and B lymphocytes, blocking polarization of $CD206^+F4/80^+Arg1^+$ MDSCs	[49], [55], [65], [72-74]
**Meflin⁺**	• Expressed on αSMA^{low}; $FAP^{+/-}$; $PDGFR\alpha^+$; $Gli1^+$ CAFs<br>• Increase vascularization and T-cell infiltration<br>• Reduce stiffness<br>• High Meflin⁺ CAFs are favorable for response to immune checkpoint blockade (ICB) and increased chemosensitivity	[150-153]
**CD271⁺/NGFR⁺**	• Induced in early PDAC stages<br>• Locate distantly from tumor core<br>• High numbers of CD271⁺ CAFs correlate with a better prognosis	[155]
**Gli1⁺**	• Gli1⁺: Activated in PSCs by Hh pathway activity	[84]
**Tumor-promoting CAFs**		
**Inflammatory CAFs (iCAFs)**	• Express low level of αSMA, production of inflammatory cytokines<br>• Secrete IL6 and LIF, among others<br>• Promote invasion of cancer cells, metastasis, immune evasion, activation of M2 macrophages<br>• Activation of JAK/STAT leading to iCAF differentiation, CD8⁺ cell exclusion, infiltration of M2 macrophages	[49], [94-99], [103-106], [110]
**Zeb1⁺**	• Stimulate RAS activity in pancreatic cancer cells supporting their migration, invasion and proliferation<br>• Probably represent a myCAF subpopulation	[77-78]
**LRRC15⁺**	• TGF-β-induced subtype of (probably) myCAFs<br>• Regulate cytotoxic T-cells<br>• Poor response to PD-L1 therapy	[50], [83]
**Saa3⁺**	• Upregulated in PDGFRα⁺ iCAFs and apCAFs<br>• Stimulate tumor growth	[47], [49], [107]
**FAP⁺/CXCL12⁺**	• Secrete CXCL12, resulting in CD8⁺ T cell exclusion<br>• Attract Tregs<br>• Cleavage of collagen, ECM modulation<br>• Support progression of PDAC and metastasis<br>• High expression is associated with a worse clinical outcome	[118], [119], [52], [122-125]
**CD10⁺/GPR77⁺**	• Release of IL6 and IL8, complement factor C5a<br>• Maintaining a niche for CSC	[113], [126]
**CD105⁺**	• Could be polarized into iCAF or myCAF<br>• Immunosuppression, reduce tumor infiltration with T-cells and DC<br>• Accelerate tumor growth, however depletion of CD105⁺ CAFs did not inhibit tumor progression in KPC	[145], [149]
**Hypoxia⁺**	• Immunosuppression<br>• Inhibition of T-cell infiltration and activity<br>• Promote infiltration with Tregs and M2 macrophages	[134], [135], [138], [141]
**Metabolic CAFs**	• Produce pyruvate and lactate, among others<br>• Provide amino acids and support tumor cell growth<br>• Autophagy in CAFs facilitates paracrine cancer growth	[162-165], [58]
**Antigen-presenting CAFs (apCAFs)**	• Expression of MHCII and CD74<br>• Lack of induction of CD4⁺ T cell proliferation<br>• Derived from mesothelial cells, express MSLN<br>• Can be activated into iCAF and myCAF<br>• Most likely immune suppressive	[47], [144], [50]
**CAFs with unknown relevance**		
**Complement-secreting CAFs (csCAFs)**	• Express complement components C3, C7, CFB, CFD, CFH, and CFI<br>• Presumed to modulate immune and inflammatory responses	[113]
**HoxB6⁺**	• Unknown functionality	[84]

Blue: Tumor-restraining CAFs; red: Tumor-promoting CAFs; grey; CAFs with unknown functionality.
